# Estimating CO_2_ emissions from international medical electives: a literature review and quantitative analysis

**DOI:** 10.1016/j.fhj.2025.100453

**Published:** 2025-07-29

**Authors:** Luke Coakham, Nihal Sogandji, Amy Stuart, Magnus Macleod, Faris Khan, Amina Ali, Christine Agbenu, Yuhui Zhou, Martin Tam, Edward Lau, Arthur Hibble, James N Smith, Anmol Arora, Charlotte Tulinius

**Affiliations:** a School of Clinical Medicine, University of Cambridge, Cambridge, United Kingdom; b Public Health Education Group, Public Health and Primary Care Unit, University of Cambridge, Cambridge, United Kingdom

**Keywords:** Sustainability, Medical school, Curriculum design, Virtual electives, Clinical education

## Abstract

**Introduction:**

Electives are short placements during medical school lasting 2–8 weeks, serving as an opportunity to engage with different healthcare systems and cultures and to travel overseas. However, amid increasing alarm about climate change, interest in the sustainability of electives and alternative elective formats are gaining attention.

**Methods:**

A scoping review of MEDLINE, Embase, ERIC, Web of Science SCOPUS, WHO Globus Index Medicus and Scielo was conducted with double-blind screening to identify previous efforts to quantify carbon costs of electives. To quantify the carbon dioxide (CO_2_) emissions of electives, we created an approach based on the fuel efficiency of aircraft used for long-haul travel, distances from the UK to popular elective destinations and the average occupancy rates of aeroplanes. These results were compared with results from seven existing resources: MyClimate, ICAO, Google Flights, C Level and EcoTree.

**Results:**

The review did not identify any previous studies estimating the environmental costs of medical student electives. All of the 7,575 records revealed by the database search were excluded following full-text screening. Our estimates of the CO_2_ emissions from round-trip flights from Heathrow Airport, London, UK to the 10 most popular elective destinations were: Australia: 2,995 kg/person, USA: 1,039 kg/person, New Zealand: 3,316 kg/person, Canada: 941 kg/person, India: 1,185 kg/person, South Africa: 1,705 kg/person, Malaysia: 1,867 kg/person, Tanzania: 1,322 kg/person, Ireland: 79 kg/person.

**Conclusion:**

This is the first study to quantify the carbon footprint of international medical electives. Our bespoke calculations, which generally agree with the results from established tools, reveal that CO_2_ emissions from international travel for electives are substantial, compared to the average annual CO_2_ emissions of 7,000 kg per person in the UK. This study provides evidence to motivate the design and delivery of alternative elective programmes.

## Highlights

• There is growing concern regarding the carbon footprint associated with face-to-face international electives.
• Virtual electives are undertaken by students and instructors who are separated in space, where instructors can teach remotely via the internet using technology such as videoconferencing and virtual reality. These virtual electives have recently appeared as an attractive alternative with the potential to contribute to improving the sustainability of medical education and student satisfaction has so far been broadly positive based on available literature.
• We performed calculations for direct round trips from the United Kingdom (UK) to the 10 most popular elective destinations for UK medical students.
• Our CO2 emissions calculator produced similar emission estimates to other calculators used, and suggests that the carbon footprint of IMEs is substantial.
• Future research should also evaluate alternative programmes, to assess whether or not virtual or local electives are considered to provide the same educational benefits as in-person electives.

• What is already known on this topic – summarise the state of scientific knowledge on this subject before you did your study and why this study needed to be done
  Before this study, the state of scientific knowledge regarding the environmental impact of international medical electives was limited, particularly in terms of quantifying the carbon emissions associated with such electives. While the importance and benefits of electives in medical education are well-documented, their environmental costs, especially the carbon footprint resulting from air travel, had not been systematically studied or quantified. This original research paper presents the first study on the environmental consequences of international medical electives, at a time when medical schools and students seek to balance educational benefits with sustainability concerns.
• What this study adds – summarise what we now know as a result of this study that we did not know before
  This study provides the first quantification of the carbon emissions associated with international medical electives, specifically focusing on air travel from the UK to popular elective destinations. The findings reveal significant variability in emissions estimates depending on the calculator used, but consistently show that electives contribute considerably to the carbon footprint of medical education. Additionally, the study suggests the potential for reducing emissions through alternative elective formats, such as local or virtual electives.
• How this study might affect research, practice or policy – summarise the implications of this study
  In practice, medical schools and students might be prompted to reconsider the necessity of international electives, exploring more sustainable alternatives that still meet educational objectives. There is a need for more research, particularly to evaluate whether alternative elective formats are sustainable, practical and provide comparable benefits to students. This study also contributes to the broader discourse on climate change by emphasising the role of the healthcare sector in global sustainability efforts.

## Introduction

A medical elective is a short placement organised and undertaken by a medical student during their degree. Electives provide an opportunity for students to experience medicine in a setting beyond their home institutions and are a compulsory part of many medical degrees worldwide. Students often have significant autonomy in arranging the elective, including the choice of medical specialty and the destination. As such, medical electives can be immensely beneficial in allowing students to hone their academic and interpersonal skills, while gaining novel experiences which may not be readily accessible in their home institutions.^1^

Medical students may choose to undertake an elective in a different country to their home institution, ie an ‘international medical elective’ (IME). IMEs offer the benefit of experiencing different cultures and healthcare systems,^2^ helping to foster international networks which may develop into professional collaborations and influence future career trajectories. However, organising these electives requires substantial time and effort, while carrying considerable financial burden associated largely with travel and accommodation.^3^ The stress of navigating an unfamiliar system and the possibility of logistical disruptions such as issues with visas or accommodation should not be underestimated. Indeed, the severe acute respiratory syndrome coronavirus 2 (SARS-CoV-2) pandemic saw a significant decline in the number of visiting programmes available for students, due to the suspension of many face-to-face clinical activities and travel disruptions.^4^ There are likely to be long-term consequences on IMEs following the SARS-CoV-2 pandemic, which include an increase in elective fees to compensate for the inability to run programmes during the pandemic, providing an advantage for students with a stronger financial background. There may also be increasing competition for places on IMEs due to a gradual reopening of programmes after the pandemic.^3^ Importantly, there is growing concern regarding the contribution of face-to-face international electives to the ‘triple planetary crisis’. This is a term which refers to the three interlinked issues humanity currently faces; climate change, pollution and biodiversity loss.^5^ Air travel is recognised as a major contributor to CO_2_ emissions and thus the triple planetary crisis, with climate change expected to negatively impact the global healthcare sector in the near future.^6^ Research into the environmental impact of international in-person electives is limited.

The aims of this review are:

1. to investigate previous attempts in literature to quantify the CO_2_ emissions from international electives
2. to devise a method to quantify the carbon footprint of these electives (and compare this with that of alternative, ‘local’ or virtual formats).

## Methods

### Literature review

#### Search databases

This scoping review was conducted in accordance with PRISMA guidelines. Searches were run on 2 June 2025 on all databases. The search strategy was intentionally broad, capturing the following databases: MEDLINE, EMBASE, ERIC, Web of Science, SCOPUS, WHO Globus Index Medicus and Scielo. A grey literature search was also conducted, searching Google, conference abstracts and proceedings. There were no restrictions on language.

#### Screening

Title and abstract screening was conducted in Rayyan.^7^ Each record was independently screened by two reviewers at the University of Cambridge, each blinded to the decision of the other reviewer, using the inclusion and exclusion criteria below. Conflicts were resolved by a third independent reviewer where necessary. Due to continuous advances in the carbon efficiency of commercial air travel and the relatively recent adoption of carbon calculators, it was felt that recently published papers would be most useful in answering the review question. Capturing results dating back to 2012 was felt to be a generous timeframe to capture over 10 years of literature.

The inclusion and exclusion criteria applied during abstract and full-text screening are provided below.

Inclusion criteria:

• Describes a short-term placement forming part of a medical school curriculum, which there is an opportunity to undertake at a different institution to the medical school.
• Provides quantification of the environmental cost of medical student elective programmes.
• Describes the environmental costs in terms of the amount of emissions, eg weight, volume, estimated cost to the economy, metric tonnes of carbon dioxide equivalent, social cost of carbon emissions (in any numerical currency).
• Published since 2012.

Exclusion criteria:

• Relates to placements undertaken by other professional groups, including qualified doctors.
• Relates to core parts of the curriculum which do not involve travel to another institution.
• Reviews, perspectives, opinions, letters that do not provide any specific quantification of environmental costs.

### Quantification of the CO_2_ emission cost of international electives

To investigate CO_2_ emissions from travel associated with international elective placements, we first used a range of established online calculators to estimate the carbon footprint from these flights. These calculators tended to produce variable results, encompassing models with a range of complexity. Furthermore, while models tended to provide high-level detail about the factors affecting the emissions calculations, the underlying methodology was not always publicly available. As a result, we compared the figures obtained from these websites results of our own standardised calculation method for the top 10 elective destinations.

#### Selection of elective destinations

We performed calculations for direct round trips from the UK to the 10 most popular elective destinations for UK medical students, as listed by The Electives Network, 2018.^8^ The 10 countries included were the UK, Australia, USA, New Zealand, Canada, India, South Africa, Malaysia, Tanzania and Ireland. Pre-pandemic data from 2018 were used to capture the most accurate picture of favoured travel destinations for UK medical students before the disruption of the SARS-CoV-2 pandemic.

#### Selection and use of online calculators

The seven calculators selected to estimate CO_2_ emissions were MyClimate,^8^ International Civil Aviation Organization (ICAO),^9^ Google Flights,^10^ C Level^11^ and EcoTree.^12^ This diverse set of calculators, ranging from emission reduction/awareness sites to airline platforms, was selected to capture a broad spectrum of different perspectives and eliminate potential biases in comprehensively assessing the carbon footprint of international medical electives.

#### Novel method for CO_2_ emission calculations

For our calculations, we used the average fuel consumption rate of a Boeing 747-400 aircraft, which is widely used in long-haul commercial flights, over a 35,000-mile journey. This rate is 19 L fuel per mile,^13^ which was used to calculate the CO_2_ emissions per mile.

This was then multiplied by the distance in miles of a straight line from London Heathrow Airport to the capital city of each country (calculated from Google Maps) to find the total CO_2_ emissions during each round trip. To calculate CO_2_ emissions per person, emissions per round trip were divided by 339 passengers. This number was based on the Boeing 747-400 being filled to 67.8% capacity, the average occupancy rate of multiple airlines with flights to and from the UK in 2022.^14^ The results are demonstrated in Table 1. Further details on the calculations are provided in Supplementary Information.

**Table 1:** CO_2_ emissions emitted per passenger (kg) on a round-trip flight to top 10 elective destinations kg according to different established calculators and the novel calculator developed.

Location (airport)	MyClimate	ICAO	Google flights	C level	EcoTree	Mean	SD	Novel method
Australia (Canberra)	7,400	1,751	2,931	6,097	4,096	**4,455**	**2,055.8**	**2,995**
USA (Washington DC))	2,400	654	861	2,115	1,544	**1,514.8**	**680.2**	**1,039**
New Zealand (Wellington)	8,400	1,974	3,308	6,750	4,516	**4,989.6**	**2,318.4**	**3,316**
Canada (Ottawa)	2,100	576	1,089	1,916	1,416	**1,419.4**	**553.2**	**941**
India (New Delhi)	2,700	552	1,162	2,413	1,732	**1,711.8**	**789.4**	**1,185**
South Africa (Cape Town)	4,000	1,089	1,785	3,470	2,412	**2,551.2**	**1,066.6**	**1,705**
Malaysia (Kuala Lumpur)	4,400	888	1,892	3,802	2,624	**2,721.2**	**1,268.4**	**1,867**
Tanzania (Dar es Salaam)	3,000	943	1,320	2,691	1,912	**1,973.2**	**782.5**	**1,322**
Ireland (Dublin)	306	101	111	295	288	**220.2**	**93.5**	**79**

The research described in this paper did not involve direct patient participation or public involvement in its design.

## Results

### Literature screening

**Fig. 1:**
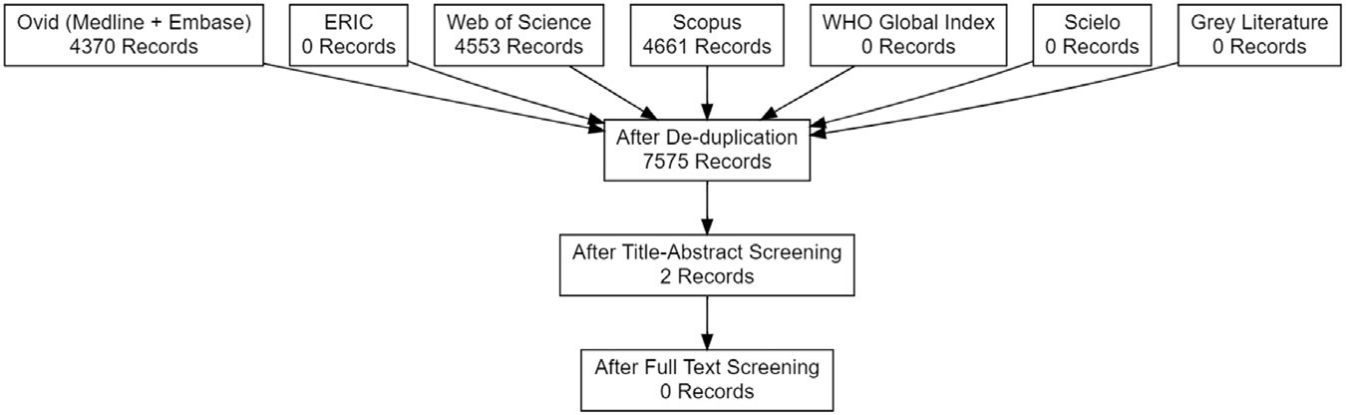
Diagram showing the inclusion of no unique studies after full-text screening, from 7,575 records screened.

After removing duplicates, the initial search produced 7,575 records. Screening of these records produced two records for full-text review. Of these, neither record was included in this scoping review after applying the inclusion and exclusion criteria (Fig. 1).

### Study characteristics

The two articles included in this scoping review after title-abstract screening, which were rejected after full-text screening, were mostly descriptive. One of these articles from Domingo *et al.*^15^ provided quantitative data on the environmental impact of USA residency programmes using virtual interviews for 2020–21 applications. Information was collected from 1,429 American medical students on their origin of travel and the mode of transportation that they would have used to attend an in-person interview. The ICAO calculator was then used to estimate flight-related greenhouse gas emissions (GHGE) in metric tonnes of carbon dioxide equivalent (MTCO2e). Approximately 98% of the GHGE savings from the switch to virtual interviews resulted from avoiding air travel, highlighting how significant air travel’s contribution is to the environmental cost of medical student travel. Although this paper did not pertain to elective programmes and therefore fell outside the scope of this review, it does highlight the environmental benefits of reducing unnecessary travel by medical students.

### Quantification of the CO_2_ emissions released during international electives

#### CO_2_ emissions per mile of flight

1 L kerosene = 0.8 kg^16^

Amount of fuel consumed by Boeing 747-400 per mile = 19 L

19 L kerosene = 15.2 kg

CO_2_ emission from 1 kg kerosene jet fuel = 3.16 kg^17^

CO_2_ emission from Boeing 747-400 in 1 mile = 3.16×15.2 = 48.032 kg

This was then used to calculate the CO_2_ emissions per person for round-trip flights from the UK to the 10 most popular elective destinations.

#### CO_2_ emissions from round-trip flights to the 10 most popular elective destinations per person

**Fig. 2:**
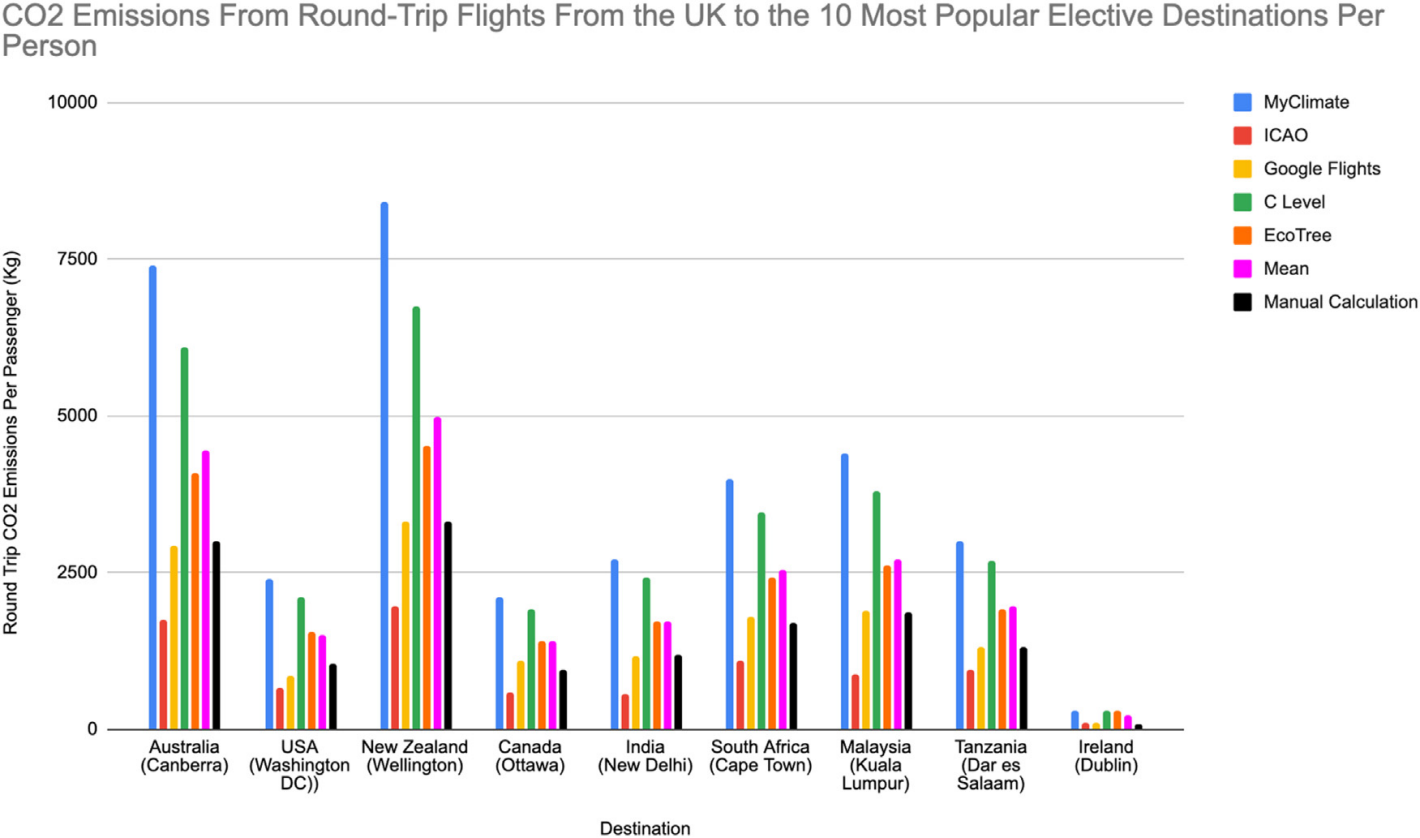
Bar chart illustrating the CO_2_ emissions from round-trip flights from the UK to the top 10 most popular elective destinations as per the Electives Network. Each colour represents the particular calculator used. Our own calculator result and mean result of all calculators used are also included as separate bars.^18^

The details of our calculations can be found in the ‘Supplementary Information’ section. The results are shown in Table 1 below alongside the results from seven online calculators and graphically illustrated in Fig. 2.

## Discussion

This is the first study to analyse the environmental costs associated with IMEs. Although carbon emissions are generally recognised as a disadvantage of in-person electives compared to virtual electives, this has not been studied in detail previously.

There is significant variation among the online calculators in the estimations of CO_2_ emissions for equivalent flights. Generally, the method by which each website calculates its results is not shared, so we were unable to establish the cause of the variation. That said, our results are all within 1 standard deviation of the mean of the established calculator results, with the exception of Ireland, which is within 2 standard deviations. Our findings suggest that for a student from the UK undertaking an elective in New Zealand, the carbon costs are equivalent to over two-thirds of the average per capita global carbon footprint for a year.^19^

### Addressing the lack of current literature on the carbon cost of medical electives

To our knowledge, this is the first paper evaluating the carbon cost of IMEs. As emphasised by our initial calculations, UK medical student electives involving international air travel are a significant contributor to CO_2_ emissions. This warrants further investigation in order to assess the environmental impact of continuing this practice and to consider more environmentally sustainable alternatives.

### Environmental impact of IMEs

Since the pre-industrial period, the mean global temperature has increased by 1.1 °C, though with significant variability across the globe.^20^ Extreme weather events and natural disasters have become more frequent, more intense and longer in duration.^21^^,^^22^ Data suggest that the increase in temperature since pre-industrial times is largely due to emissions of greenhouse gases from human activity.^23^ Carbon emissions from international travel for tourism are expected to increase 45% by 2030 relative to levels from 2016.^24^ Avoiding a single flight can be equivalent to going car-free for a year or the amount of carbon absorbed by an acre of forest in a year.^25^

There is no doubt that the triple planetary crisis has caused, and will continue to create, healthcare challenges globally. Many populations across the world are already affected by malnutrition, spread of infectious diseases and natural disasters related to shifts in climate patterns. Changing climate patterns have increased exposure to infectious diseases by allowing easier transmission of air- and water-borne microorganisms and have also been linked to cardiovascular disease, mental illnesses and premature death.^26^ Furthermore, extreme weather events also impact access to healthcare services, and tropical countries, many of whom are low- or middle-income countries with already limited access to certain services, have suffered disproportionately from the climate change burden to date.

As seen from our calculations, the carbon dioxide emission from a single round trip to popular elective destinations is significant. To provide some context to the estimated carbon cost, Klower *et al.*^27^ found that attendance at a large academic conference in California, USA, emitted the equivalent of the average weekly emissions of Edinburgh, UK, roughly 80,000 tonnes of CO_2_. This conference hosted around 28,000 delegates travelling a total of 285 million kilometres. While the number of medical students graduating in the UK in 2022 was 10,543^28^ and many of them are unlikely to undertake IMEs, the carbon cost of IMEs is likely to be similar to the average weekly emissions of a smaller city than Edinburgh. At an individual level, the average carbon cost per person per year in the UK, is estimated to be approximately 7,000 kg CO_2_.^29^

Furthermore, if we compare the carbon emissions released during a round-trip flight from the UK to Canberra, Australia using our own calculations, an individual would release around 3 tonnes CO_2_ equivalent, which is just below half of the average annual emissions of an individual in the UK according to the Department for Environment, Food and Rural Affairs (DEFRA).^30^

### Other factors to consider before embarking on IMEs

There are undoubtedly many benefits to carrying out IMEs. These include the opportunity to work with people from culturally, linguistically and socioeconomically diverse backgrounds^30^ and to observe a plethora of different medical conditions that students may have otherwise never come across. Through a focus on equity, autonomy, solidarity and participation, international collaborations can facilitate the sharing of knowledge and expertise globally.^31^

For many, elective placements are integral to the formation of a professional identity – by observing and appreciating differences in practice and the context in which international doctors practise, students may be better able to reflect on their own attitudes and habits and how these are shaped by the environment in which they have trained. This in turn helps them to be well prepared to practise in an increasingly globalised world.^32^

Additionally, medical students travelling abroad for IMEs are likely to encounter the effects of climate change first hand, and there is evidence to suggest that ‘experiential learning’ can lead to greater awareness of climate change.^33^

However, IMEs do have some other potential disadvantages worth considering. These include the substantial financial costs of international travel, accommodation, and medical placement fees. As an example, Beckschulte *et al.*^34^ found the median 4-week elective fee alone to be €364.15, and data suggest that fees are rising substantially after the SARS-CoV-2 pandemic.^34^ This significant financial barrier puts students with insufficient funding at a disadvantage to their peers and potentially exacerbates the inequalities between medical students from different socioeconomic backgrounds.

IMEs can also present a variety of cultural, social and moral issues for medical students. They may feel under pressure to practise outside their competencies or struggle to work in an environment that they feel falls below the standard of care in the UK. International electives can be a very lonely time for students, especially if they are travelling without friends to an unfamiliar country or culture^35^ or do not speak the local language.

More research is needed into the benefits the recipient community receives during IMEs. There is a risk that the power imbalance between students from high-income countries and their host communities could result in one-sided relationships which only benefit the visiting student or institution.^36^

### The rise of virtual electives following the SARS-CoV-2 pandemic and other solutions to the carbon cost of medical electives

The SARS-CoV-2 pandemic in 2020 led to government-enforced lockdowns of varying severity across the globe.^37^ For UK medical students, these conditions prevented international and often even local travel for medical student electives,^38^ the vast majority of which were cancelled by April 2020.

Sustainability concerns and the challenges experienced during the SARS-CoV-2 pandemic have brought forward the need for innovative thinking regarding medical education, including medical electives. Virtual electives are undertaken by students and instructors who are separated in space, where instructors can teach remotely via the internet using technology such as videoconferencing and virtual reality. These virtual electives have recently appeared as an attractive alternative with the potential to contribute to improving the sustainability of medical education, and student satisfaction has so far been broadly positive based on available literature.^39^, ^40^, ^41^ However, more research is required to investigate how translatable clinical experiences can be when moved to a virtual format.

Another solution for medical students who may wish to reduce their ‘elective carbon footprint’ may be to carry out ‘local’ electives within the UK, perhaps in a different region to the students’ parent medical school. This would of course involve significantly less CO_2_ emission related to air travel, but would also likely not expose students to the same range of different medical conditions and richness of experience that students may come across on an IME. Medical schools could also consider discussions around the carbon cost of air travel at elective talks, and discuss alternatives such as ‘local’, hybrid and virtual electives in these talks. Finally, we acknowledge that institutionally coordinated offsetting schemes could serve as an interim measure. However, we emphasise that offsetting should not be positioned as a substitute for emission reduction at the source. Moreover, given the substantial financial burden already placed on medical students undertaking electives, it would likely be unrealistic for offsetting initiatives to rely on individual student contributions without financial support from institutions.

### Limitations of this review

The calculations of the CO_2_ emissions associated with travel for in-person electives exclusively focused on direct round trips, which may not reflect real-life practice for many destinations. We also limited our focus to the Boeing 747-400 aircraft since it is extensively used for long-haul flights, and this may have implications for the accuracy of our calculations for shorter journeys, eg the round trip to Ireland.

We do not have clear data on the number of UK medical students who embark on IMEs, and while we did find information on the top 10 most popular elective destinations of medical students in the UK from the Electives Network,^18^ the data that underpinned this list were not shared. Unfortunately we could not find a more accurate list of the top 10 elective destinations in the literature. We also note that there are access restrictions for ‘The Electives Network’ and therefore this is a source of bias in our review. We also note that elective databases are limited in their accuracy when it comes to documented elective destinations.^42^ Furthermore, our calculations were based on the most popular IME destinations for UK medical students, and there is literature to suggest that medical students from other European countries differ in their preference of destination. For example, Storz *et al.*^43^ found that over 50% of German-speaking medical students carry out electives in industrialised European countries.^43^

There are a number of variables that we did not calculate in this paper, as we decided to focus our calculations on the CO_2_ emissions associated with international travel. However, it is worth considering other sources of carbon emissions during IMEs, such as extra trips taken by students while abroad, and therefore our calculations are likely to underestimate CO_2_ emissions associated with IMEs. Similarly, we did not calculate any carbon cost for domestic travel for students completing their elective within the UK.

### Need for more research in the area

To conclude, the lack of published literature quantifying the carbon footprint of international medical electives illustrates that this is currently an unexplored area of research. Our CO_2_ emissions calculator produced similar emission estimates to other calculators used, and suggests that the carbon footprint of IMEs is substantial. Virtual electives may be a popular and environmentally sustainable alternative, based on the literature currently available.

Future directions of research should include extensive investigation into the overall CO_2_ emissions produced by medical students on elective each year, using formal elective destination reports from every UK medical school. Future research should also evaluate alternative programmes, to assess whether or not virtual or local electives are considered to provide the same educational benefits as in-person electives.

## Ethics approval and consent to participate

Not applicable.

## Funding

This research did not receive any specific grant from funding agencies in the public, commercial, or not-for-profit sectors.

## Data availability

The data that supports the findings of this study are available in the supplementary material of this article.

## CRediT authorship contribution statement

**Luke Coakham:** Writing – original draft, Visualization, Methodology, Investigation. **Nihal Sogandji:** Writing – original draft, Visualization, Methodology, Investigation. **Amy Stuart:** Writing – review & editing, Investigation, Data curation. **Magnus Macleod:** Writing – review & editing, Investigation, Data curation. **Faris Khan:** Writing – review & editing, Investigation, Data curation. **Amina Ali:** Writing – review & editing, Investigation, Data curation. **Christine Agbenu:** Writing – review & editing, Investigation, Data curation. **Yuhui Zhou:** Writing – review & editing, Investigation, Data curation. **Martin Tam:** Writing – review & editing, Investigation, Data curation. **Edward Lau:** Writing – review & editing, Project administration, Investigation, Data curation, Conceptualization. **Arthur Hibble:** Writing – review & editing, Supervision, Project administration, Methodology, Investigation, Conceptualization. **James N Smith:** Writing – review & editing, Supervision, Project administration, Methodology, Investigation, Conceptualization. **Anmol Arora:** Writing – review & editing, Visualization, Supervision, Project administration, Methodology, Investigation, Data curation, Conceptualization. **Charlotte Tulinius:** Writing – review & editing, Supervision, Project administration, Methodology, Investigation, Conceptualization.

## Declaration of competing interest

The authors declare the following financial interests/personal relationships which may be considered as potential competing interests:

Anmol Arora is an Associate Editor of the *Future Healthcare Journal*. If there are other authors, they declare that they have no known competing financial interests or personal relationships that could have appeared to influence the work reported in this paper.

## Appendix Supplementary materials

Image, application 1
