# Supplementary material for: Estimating CO2 emissions from international medical electives: a literature review and quantitative analysis
Source: Future Healthc J. 2025 Jul 29;12(3):100453. doi: 10.1016/j.fhj.2025.100453 (PMC12395515; doi:10.1016/j.fhj.2025.100453)
Supplement: Supplementary file 1 [file mmc1.docx]

**Supplementary Information**

**Destinations and Calculations:**

Emission calculations were based on distances between London Heathrow and the major international airport of the capital city of each destination country. For South Africa, Cape Town was chosen as the destination city because it has the largest population of the three capital cities [14]. The only exception to the choice of destination airport was for Tanzania; despite being in Tanzania’s capital city, Dodoma’s airport only runs domestic flights. Instead, we used the airport in Dar es Salaam, the country’s most populous city[15]. For the remaining calculators,, C Level and EcoTree, the only information entered into the calculators were the origin and destination airports. Some calculators required additional data input. When using MyClimate, the Boeing 787 aeroplane was used to calculate emissions because it was not possible to select the Boeing 747 and the Boeing 787 was the closest option to the Boeing 747 in terms of fuel per seat[16]. For the ICAO calculator, we were required to specify layover airports for countries to which there are no direct flights. Where this occurred, we used layover routes to the final destination deemed ‘fastest’ by Skyscanner[17]. For the Google Flight calculator, we used emissions data from flights listed as ‘average emissions’. For numerous countries on our list, there are several common elective destinations within the country, and we have only calculated carbon emissions to the major international airport of the capital city. We measured distances in a straight line (‘as the crow flies’) using Google Maps. This simplification will not fully capture specific flight paths or the need for more than one flight journey to reach the specified destination, and may well produce an underestimation of the distance.

1. **London Heathrow Airport, United Kingdom:**

○ Distance = 0 miles

○ Emissions per person = 0 kg CO_2_

1. **Canberra Airport, Australia:**

○ Distance = 10,569 miles

○ Round-trip distance = 21,138 miles

○ Emissions per round trip (to the nearest kg) =

○ 21,138miles × 48.032 kg/mile = 1,015,300 kg

○ Emissions per person (to the nearest kg) =

○ 1,015,300 kg/339 passengers = 2,995 kg/person

1. **Washington Dulles International Airport, USA:**

○ Distance = 3,667 miles

○ Round-trip distance = 7,334 miles

○ Emissions per round trip (to the nearest kg) =

○ 7,334 miles × 48.032 kg/mile = 352,267 kg

○ Emissions per person (to the nearest kg) =

○ 352,267 kg/339 passengers = 1,039 kg/person

1. **Wellington International Airport, New Zealand:**

○ Distance = 11,701 miles

○ Round-trip distance = 23,402 miles.

○ Emissions per round trip (to the nearest kg) =

○ 23,402 miles × 48.032 kg/mile = 1,124,045 kg

○ Emissions per person (to the nearest kg) =

○ 1,124,045 kg/339 passengers = 3,316 kg/person

1. **Ottawa International Airport, Canada:**

○ Distance = 3,322 miles

○ Round-trip distance = 6,644 miles

○ Emissions per round trip (to the nearest kg) =

○ 6,644 miles × 48.032 kg/mile = 319,125 kg

○ Emissions per person (to the nearest kg) =

○ 319,125 kg / 339 passengers = 941 kg/person

1. **Delhi Airport, India:**

○ Distance = 4,183 miles

○ Round-trip distance = 8,366 miles

○ Emissions per person (to the nearest kg) =

○ 8,366 miles × 48.032 kg/mile = 401,836 kg

○ Emissions per person (to the nearest kg) =

○ 401,836 kg/339 passengers = 1185 kg/person.

1. **Cape Town International Airport, South Africa:**

○ Distance = 6,016 miles

○ Round-trip distance = 12,032 miles

○ Emissions per round trip (to the nearest kg) =

○ 12,032 miles × 48.032 kg/mile = 577,921 kg

○ Emissions per person (to the nearest kg) =

○ 577,921 kg/339 passengers = 1705 kg/person

1. **Kuala Lumpur International Airport, Malaysia:**

○ Distance = 6,590 miles

○ Round-trip distance of 13,180 miles.

○ Emissions per round trip (to the nearest kg) =

○ 13,180 miles × 48.032 kg/mile = 633,062 kg

○ Emissions per person (to the nearest kg) =

○ 633,062 kg/339 passengers = 1867 kg/person

1. **Julius Nyere International Airport, Dar es Salaam, Tanzania:**

○ Distance = 4,664 miles

○ Round-trip distance = 9,328 miles

○ Emissions per round trip (to the nearest kg) =

○ 9,328 miles × 48.032 kg/mile = 448,042 kg

○ Emissions per person (to the nearest kg) =

○ 448,042 kg/339 passengers = 1322 kg/person

1. **Dublin Airport, Ireland:**

○ Distance = 279 miles

○ Round-trip distance = 558 miles

○ Emissions per round trip (to the nearest kg) =

○ 558 miles × 48.032 kg/mile = 26,802 kg

○ Emissions per person (to the nearest kg) =

○ 26,802 kg/339 passengers = 79 kg/person

**Search Strategy Strings:**

Screening Completed On 29 August at 18:28

Using Google Chrome on a MacBook Pro Running Ventura 13.3.1 (a)

**Medline:**

**Ovid MEDLINE(R) and Epub Ahead of Print, In-Process, In-Data-Review & Other Non-Indexed Citations, Daily and Versions <1946 to August 28, 2023>**

1 (placement* or elective* or attachment* or rotation* or intern* or clerkship*).ti,ab,kw,kf. 1662787

2 ((student or trainee or undergraduate*) adj (doctor* or medic* or clinical)).ti,ab,kw,kf. 10084

3 exp Education, Medical, Undergraduate/ or exp Students, Medical/ 59770

4 2 or 3 63970

5 1 and 4 12827

6 exp Environmental Monitoring/ or exp "Conservation of Natural Resources"/ or exp Air Pollution/ or Greenhouse Effect/ 332177

7 (carbon or sustain* or emission* or green or pollut* or environment* or greenhouse).ti,ab,kw,kf. 2641788

8 6 or 7 2784094

9 5 and 8 1504

10 limit 9 to yr="2012 -Current" 1078

1078 results

**Embase:**

**Embase <1974 to 2023 August 28>**

1 (placement* or elective* or attachment* or rotation* or intern* or clerkship).ti,ab. 2225092

2 ((student or trainee or undergraduate*) adj (doctor* or medic* or clinical)).ti,ab. 10494

3 exp *medical education/ 174638

4 exp *medical student/ 35245

5 2 or 3 or 4 203462

6 (carbon or sustain* or emission* or green or pollut* or environment* or greenhouse).ti,ab. 3010585

7 exp *carbon emission/ or exp *greenhouse effect/ or exp *carbon footprint/ 11061

8 6 or 7 3013382

9 1 and 5 and 8 3347

10 limit 9 to yr="2012 -Current" 2198

2198 results

**ERIC**

(placement* or elective* or attachment* or rotation* or intern* or clerkship*) AND (((student or trainee or undergraduate*) adj (doctor* or medic* or clinical)) OR (exp Education, Medical, Undergraduate/ or exp Students, Medical/)) AND ((exp Environmental Monitoring/ or exp "Conservation of Natural Resources"/ or exp Air Pollution/ or exp Greenhouse Effect/) OR (carbon or sustain* or emission* or green or pollut* or environment* or greenhouse)) pubyearmin:2012

2 results

**WebOfScience:**

# Searches:

11: (((TI=( (placement* or elective* or attachment* or rotation* or intern* or clerkship*))) OR AB=( (placement* or elective* or attachment* or rotation* or intern* or clerkship*))) OR AK=( (placement* or elective* or attachment* or rotation* or intern* or clerkship*)))

Date Run: Tue Aug 29 2023 18:33:50 GMT+0100 (British Summer Time) Results: 3604208

12: (((TI=(((student or trainee or undergraduate*) adj (doctor* or medic* or clinical)))) OR AB=(((student or trainee or undergraduate*) adj (doctor* or medic* or clinical)))) OR AK=(((student or trainee or undergraduate*) adj (doctor* or medic* or clinical))))

Date Run: Tue Aug 29 2023 18:34:10 GMT+0100 (British Summer Time) Results: 159671

13: ALL=(exp Education, Medical, Undergraduate or exp Students, Medical)

Date Run: Tue Aug 29 2023 18:34:34 GMT+0100 (British Summer Time) Results: 1976

14: #12 OR#13

Date Run: Tue Aug 29 2023 18:36:37 GMT+0100 (British Summer Time) Results: 161106

15: #11 AND #14

Date Run: Tue Aug 29 2023 18:37:12 GMT+0100 (British Summer Time) Results: 33528

16: ALL=(exp Environmental Monitoring or exp "Conservation of Natural Resources" or exp Air Pollution or exp Greenhouse Effect)

Date Run: Tue Aug 29 2023 18:37:32 GMT+0100 (British Summer Time) Results: 2983

17: (((TI=((carbon or sustain* or emission* or green or pollut* or environment* or greenhouse))) OR AB=((carbon or sustain* or emission* or green or pollut* or environment* or greenhouse))) OR AK=((carbon or sustain* or emission* or green or pollut* or environment* or greenhouse)))

Date Run: Tue Aug 29 2023 18:37:49 GMT+0100 (British Summer Time) Results: 7540180

18: #16 OR #17

Date Run: Tue Aug 29 2023 18:38:31 GMT+0100 (British Summer Time) Results: 7541078

19: #15 AND #18

Date Run: Tue Aug 29 2023 18:39:32 GMT+0100 (British Summer Time) Results: 4475

20: #15 AND #18 Timespan: 2012-01-01 to 2023-08-29

Date Run: Tue Aug 29 2023 18:41:47 GMT+0100 (British Summer Time) Results: 3491

Index date: From 2012-01-01 to 2023-08-29

3491 results

**SCOPUS:**

( TITLE-ABS ( placement* OR elective* OR attachment* OR rotation* OR intern* OR clerkship* ) ) AND ( ( TITLE-ABS ( ( student OR trainee OR undergraduate* ) W/0 ( doctor* OR medic* OR clinical ) ) ) OR ( TITLE-ABS-KEY ( "*medical education" ) ) OR ( TITLE-ABS-KEY ( "*medical student" ) ) ) AND ( ( TITLE-ABS-KEY ( "*carbon emission" OR "*greenhouse effect" OR "*carbon footprint" ) ) OR ( TITLE-ABS ( carbon OR sustain* OR emission* OR green OR pollut* OR environment* OR greenhouse ) ) ) AND PUBYEAR > 2011 AND PUBYEAR < 2024

3490 results

**WHO Globus Index Medicus:**

(tw:((placement* or elective* or attachment* or rotation* or intern* or clerkship*))) AND (tw:(("student doctor*" OR "student medic*" OR "trainee doctor*" OR "trainee medic*" OR "undergraduate* doctor*" OR "undergraduate* medic*" OR "clinical student" OR "clinical trainee" OR "clinical undergraduate*" OR "*medical education" OR "*medical student")) AND (tw:((carbon or sustain* or emission* or green or pollut* or environment* or greenhouse OR "*carbon emission" OR "*greenhouse effect" OR "*carbon footprint")) DA:2012$ OR DA:2013$ OR DA:2014$ OR DA:2015$ OR DA:2016$ OR DA:2017$ OR DA:2018$ OR DA:2019$ OR DA:2020$ OR DA:2021$ OR DA:2022$ OR DA:2023$

0 results

**Scielo:**

((placement* or elective* or attachment* or rotation* or intern* or clerkship*)) AND (("student doctor*" OR "student medic*" OR "trainee doctor*" OR "trainee medic*" OR "undergraduate* doctor*" OR "undergraduate* medic*" OR "clinical student" OR "clinical trainee" OR "clinical undergraduate*" OR "*medical education" OR "*medical student")) AND ((carbon or sustain* or emission* or green or pollut* or environment* or greenhouse OR "*carbon emission" OR "*greenhouse effect" OR "*carbon footprint")) AND (year_cluster:(2012-2023))

0 results
